# Metabolic profiling of zebrafish embryo development from blastula period to early larval stages

**DOI:** 10.1371/journal.pone.0213661

**Published:** 2019-05-14

**Authors:** Sundeep S. Dhillon, Frida Torell, Magdalena Donten, Katrin Lundstedt-Enkel, Kate Bennett, Stefan Rännar, Johan Trygg, Torbjörn Lundstedt

**Affiliations:** 1 School of Clinical and Experimental Medicine, College of Medical and Dental Sciences, University of Birmingham, Birmingham, United Kingdom; 2 Computational Life Science Cluster (CLiC), Department of Chemistry, Umeå University, Umeå, Sweden; 3 Accelerator Lab (ACL), Karlsruhe Institute of Technology, Karlsruhe, Germany; 4 AcureOmics, Umeå, Sweden; 5 Environmental toxicology, Organismal Biology, Uppsala University, Uppsala, Sweden; 6 Corporate Research, Sartorius AG, Göttingen, Germany; 7 Deparment of Pharmaceutical Bioscience, BMC, Uppsala University, Uppsala, Sweden; Instituto de Investigacion Sanitaria INCLIVA, SPAIN

## Abstract

The zebrafish embryo is a popular model for drug screening, disease modelling and molecular genetics. In this study, samples were obtained from zebrafish at different developmental stages. The stages that were chosen were 3/4, 4/5, 24, 48, 72 and 96 hours post fertilization (hpf). Each sample included fifty embryos. The samples were analysed using gas chromatography time-of-flight mass spectrometry (GC-TOF-MS). Principle component analysis (PCA) was applied to get an overview of the data and orthogonal projection to latent structure discriminant analysis (OPLS-DA) was utilised to discriminate between the developmental stages. In this way, changes in metabolite profiles during vertebrate development could be identified. Using a GC-TOF-MS metabolomics approach it was found that nucleotides and metabolic fuel (glucose) were elevated at early stages of embryogenesis, whereas at later stages amino acids and intermediates in the Krebs cycle were abundant. This agrees with zebrafish developmental biology, as organs such as the liver and pancreas develop at later stages. Thus, metabolomics of zebrafish embryos offers a unique opportunity to investigate large scale changes in metabolic processes during important developmental stages in vertebrate development. In terms of stability of the metabolic profile and viability of the embryos, it was concluded at 72 hpf was a suitable time point for the use of zebrafish as a model system in numerous scientific applications.

## Introduction

The zebrafish (*Danio rerio*) has emerged as a successful scientific platform for studies of metabolism and metabolic diseases [1]. The embryonic developmental stages of zebrafish have been characterized by Kimmel *et al*. [2], while Parichy *et al*. [3] described the normal table of post-embryonic zebrafish development. These two excellent studies make it possible to assign individual zebrafish to particular developmental stages, and describe the developmental changes in a variety of anatomical traits and how these traits vary over the zebrafish life cycle.

The advantages of zebrafish studies are well established and include transparency of the fertilized embryos, rapid external development, tractable genetics, generation of embryos in large numbers and the availability of a large plethora of established scientific approaches. In addition, much of zebrafish physiology and anatomy is homologous to mammalian physiology and anatomy [4]. The genetic similarities with humans is another advantage of the zebrafish as an animal model [5]. In a study of cyanide exposure Nath *et al*. have shown that metabolic changes parallel human responses to cyanide exposure, suggesting that metabolic changes are conserved between the two species [6].

**Fig 1:**
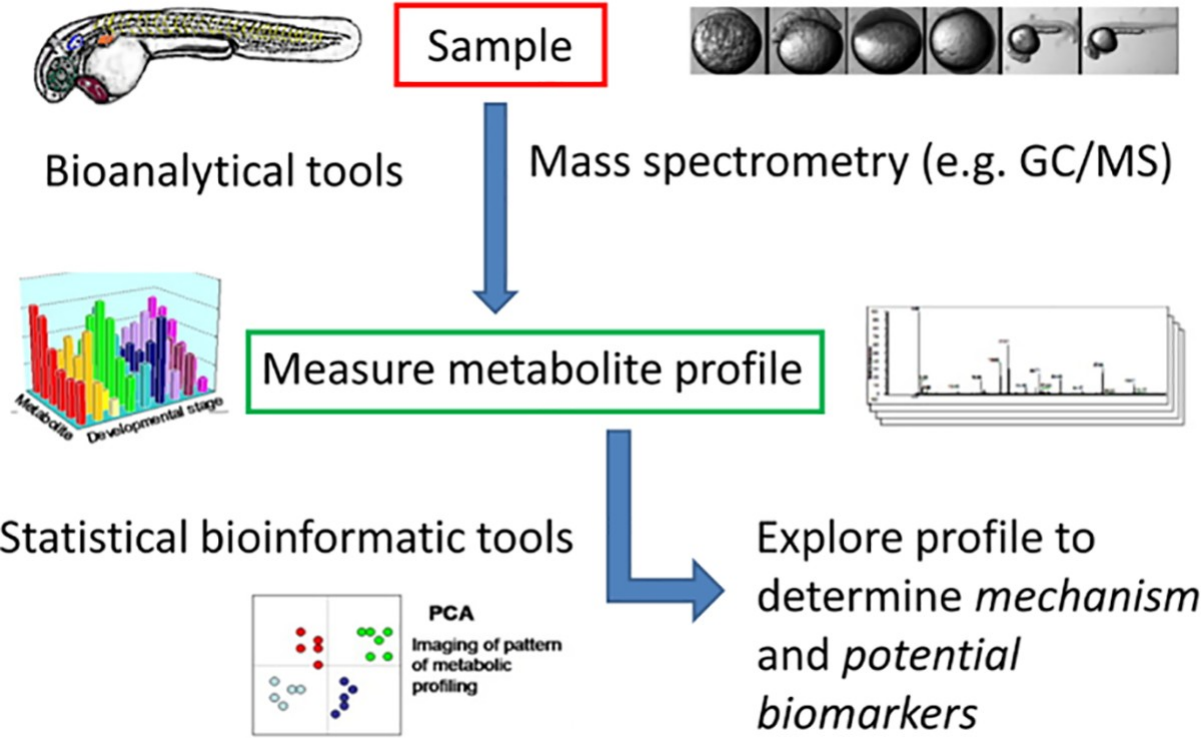
Process for metabolic profiling of zebrafish embryos.

Metabolomics enables a wide range of metabolites associated with an organism to be identified. The identified metabolites reflects the current state of an organism at a given time point. Using a metabolomics approach, changes in metabolites during development, progression of a disease or in response to chemical insult can be followed. Most of the research to date has focused on using other ‘omics’ technologies for zebrafish research. However, a metabolomics approach for zebrafish research is now starting to be developed. For example, metabolic profiling of zebrafish embryos between 0–48 hours post fertilisation (hpf) revealed significant developmental changes in the metabolome as the embryos aged [7–9]. Furthermore, metabolomic data from zebrafish could be correlated to transcriptome data to provide information on novel biological functions of genes in metabolic processes [10]. Thus, the zebrafish embryo presents an exciting opportunity to investigate metabolic processes during vertebrate development and could aid in the identification of novel biomarkers of disease or drug toxicities, see Fig 1.

As events of embryogenesis become better understood, many zebrafish researchers have begun to focus on processes that occur after embryogenesis [11]. Other researchers focus solely on unhatched larvae [7]. We identified a knowledge gap regarding both the metabolic differences between hatched and unhatched larvae and metabolic differences associated with later stages of embryogenesis to early postembryonic development. The present study was performed to identify the developmental stage where zebrafish metabolism is stable enough to be used as an animal model. In this study, we used gas chromatography time of flight mass spectrometry (GC-TOF-MS) to obtain metabolic profiles representative of different developmental stages. The developmental stages that were studied were early developmental stages (including 3/4 hpf and 4/5 hpf) and later developmental stages (including 24 hpf, 48 hpf, 72 hpf and 96 hpf). By dividing the samples in this way we were able to study key developmental stages i.e. cleavage (3/4 hpf), pharyngula (24 hpf), hatching (48 hpf), early larva (72 hpf) and later larva (96 hpf) [2]. Multivariate data analysis was used to assess differences from one developmental stage to the next. Principle component analysis (PCA) was applied to get an overview of the data and orthogonal projection to latent structure discriminant analysis (OPLS-DA) was utilised to discriminate between the developmental stages as well as between hatched and unhatched larvae. In this way, the metabolic processes during vertebrate development could be identified.

## Materials and methods

### Animal husbandry

#### Animals

Adult zebrafish from the AB* wild-type strain were kept in group sizes of approximately 15 fish in a ZEBTEC zebrafish housing system (Tecniplast, Italy). The water quality, water hardness and conductivity were controlled daily and the pH of the water was kept at 7–7.5. The fish were fed *ad libitum* twice daily, once with live food (*Artemia*) and once with dry food (TetraMin tropical flakes). The temperature in the fish room was maintained at 28°C and the light and dark cycle was controlled automatically with 14 hours light/ 10 hours darkness.

#### Zebrafish breeding and embryo collection

The day before embryos were required pairs consisting of 1 male and 1 female zebrafish were placed in breeding cages (Tecniplast, Italy). The breeding cages were equipped with spawning trays (Tecniplast, Italy) to collect the embryos and a divider was placed in the breeding cage separating the male from the female. The divider was removed the next morning. After 1 hour fertilized embryos were collected and transferred to new Petri dishes containing embryo medium (E3), which was prepared from E3 stock solution (5 mM sodium chloride, 0.33 mM calcium chloride, 0.33 mM magnesium sulphate and 0.17 mM potassium chloride) by diluting 1 in 60 with distilled water. Embryos were kept and raised in a 28°C incubator.

#### Zebrafish sample acquisition and preparation

Embryos were staged based on the staging series outlined by Kimmel *et al*. (1995) [2] and divided into different groups based on the stages chosen for metabolomics analysis. The stages that were chosen were 3/4 hpf, 4/5 hpf, 24 hpf, 48 hpf, 72 hpf and 96 hpf. Embryos at each particular stage were transferred to 1.5 mL eppendorf tubes giving 50 embryos in each tube. The liquid from each tube was then removed as much as possible using a fine tip polyethylene transfer pipette (Thermo Fisher Scientific, UK). The tubes were snap frozen by dipping them in liquid nitrogen for 30 seconds and the tubes were subsequently stored at -80°C.

Additionally, embryos were also collected at 24 hpf and were dechorionated by adding pronase enzyme solution at 1 mg/mL which was prepared by dissolving *Streptomyces griseus* Type XIV powder (Sigma-Aldrich, Poole, UK) in E3 embryo medium. After adding the pronase enzyme solution, once one third of the embryos came out of the chorions the embryos were thoroughly washed three times with 500 mL of E3 embryo medium before being transferred to a new Petri dish containing fresh E3 embryo medium. Embryos were immediately sampled after this step. Embryos without pigment formation at 48 hpf were also chosen for metabolomic analysis. These embryos were raised in 0.002% N-Phenylthiourea (PTU) (Sigma-Aldrich, Poole, UK) from 24 hpf onwards to prevent the formation of pigment and were sampled at 48 hpf.

### Metabolomic analysis

#### Materials

All standard reagents were of analytical grade or equivalent and obtained from Sigma-Aldrich (St Louis, MO, USA), Merck (Darmstadt, Germany) and J.T. Baker (Phillipsburg, NJ, USA). N-Methyl-N-trimethylsilyltrifluoroacetamide (MSTFA) plus 1% trimethylchlorosilane (TMCS) and pyridine were obtained from Thermo Fisher Scientific (Rockford, IL, USA). The stable isotope-labelled internal standards, [^13^C_5_]-proline, [^2^H_4_]-succinic acid, [^13^C_5_,^15^N]-glutamic acid, [1,2,3-^13^C_3_]-myristic acid, [^2^H_7_]-cholesterol and [13C4]-disodium α-ketoglutarate were purchased from Cambridge Isotope Laboratories (Andover, MA); [^13^C_6_]-glucose, [^13^C_12_]-sucrose, [^13^C_4_]-hexadecanoic acid and [^2^H_4_]-putrescine were purchased from Campro (Veenendaal, The Netherlands) and [^2^H_6_]-salicylic acid was obtained from Icon (Summit, NJ). Stock solutions of each internal standard were prepared in either Milli-Q water or methanol to a concentration of 0.5 μg/μL and stored at 4°C.

#### Metabolite extraction

For metabolite extraction, 1000 μl extraction buffer (methanol:H_2_O, 8:2, v/v), containing all eleven labelled internal standards (7 ng/μl), was added to each sample (50 embryos per tube). A 3 mm tungsten carbide bead (Retsch GmbH & Co. KG, Haan, Germany) was also added to each tube prior to mixing to increase extraction efficiency. All samples were extracted using a MM 400 Vibration Mill (Retsch GmbH & Co. KG, Haan, Germany) at a frequency of 30 Hz for 2 min. The beads were removed and the samples were centrifuged at 18 620 g for 15 min at 4°C. After centrifugation, 200 μl supernatant was transferred to a GC microvial (Chromacol, Herts, UK) and evaporated to dryness using a SpeedVac concentrator (Savant Instrument, Framingdale, NY, USA). All samples were stored at -80°C until derivatisation.

#### Derivatisation of samples

For GC-MS derivatisation, 30 μL methoxyamine (15 μg/μL) in pyridine was added to each GC vial and the samples were shaken vigorously for 10 min. Methoxymation was performed at room temperature for 17 h, followed by the addition of 30 μL MSTFA with 1% TMCS to each sample. Samples were vortexed briefly and left at room temperature for 1 h to allow silylation to occur. Finally, 30 μL heptane (containing 15 ng/μL methyl stearate as an internal standard) was added and vortexed briefly for 10 s ready for GC-MS analysis.

#### GC-TOF-MS analysis

A volume of 1 μL of each derivatised sample was injected splitless by a CTC Combi Pal autosampler (CTC Analytics AG, Zwingen, Switzerland) into an Agilent 6980 GC equipped with a 10 m X 0.18 mm i.d. fused-silica capillary column chemically bonded with 0.18-um DB 5-MS stationary phase (J&W Scientific Folsom, CA). The injector temperature was set to 270°C. Helium was used as the carrier gas at a constant flow rate of 1 mL min^-1^ through the column. For every analysis, the purge time was set to 60 s at a purge flow rate of 20 mL min^-1^ and an equilibrium time of 1 min. The column temperature was held initially at 70°C for 2 min, then increased to 320°C at a rate of 30°C min^-1^, where it was held for 2 min. The column effluent was introduced into the ion source of a Pegasus III time-of-flight mass spectrometer (Leco Corp., St Joseph, MI). The ion source and transfer line temperatures were set to 200°C and 250°C, respectively. Ions were generated by a 70-ev electron beam at a current of 2.0 mA. Masses were acquired in the mass range 50–800 m/z at a rate of 30 spectra s^-1^. The acceleration voltage was turned on after a solvent delay of 150 s.

#### Data analysis of all samples analysed by GC/MS

Non-processed MS files from GC/TOFMS analysis were exported in NetCDF format to MATLAB software 7.11 (Mathworks, Natick, MA), where all data pre-treatment procedures, such as baseline correction, chromatogram alignment, time-window setting and multivariate curve resolution (MCR) [12, 13] were performed using custom scripts. For the identification of metabolites, NIST MS Search 2.0 software was used to compare the mass spectra of all detected compounds with spectra in the NIST library 2.0, the in-house mass spectra library established by Umeå Plant Science Centre and the mass spectra library maintained by the Max Planck Institute in Golm (http://csbdb.mpimp-golm.mpg.de/csbdb/gmd/gmd.html). A retention index comparison was performed, with a retention index deviation <+-10 (in addition to a high spectral match) resulting in a positive ID. LECO ChromaTOF software v4.32 (Leco Corp., St Joseph, MI) was also used as an additional tool for metabolite identification. Automatic peak detection and mass spectrum deconvolution with ChromaTof software were performed using a peak width set to 2 s.

The data were normalised using all 11 internal standards (eluting over the whole chromatographic time range). To obtain accurate peak areas for the internal standards, 2 unique masses for each compound were specified and the samples were reprocessed using an in-house MATLAB 7.11 based script. The resulting dataset with relative metabolite concentrations is available as supporting information S1 Data.

#### Multivariate data analysis

A PCA, using these peak areas, was calculated and the T-score value for each sample was used to normalise the resolved data by dividing the peak areas of each sample with the corresponding score value. Multivariate analysis was performed with SIMCA 14 software (Umetrics AB, Umeå, Sweden). PCA [14, 15] was used for metabolomics data overview, and OPLS-DA [16, 17] was performed to compare metabolite patterns between different stages. UV scaling was used for both model types. OPLS-DA p(corr) values were used to evaluate the contribution of each metabolite between group separation [18]. The significance of these findings were evaluated by t-tests. Variance was checked by means of F-test. P-values were calculated by applying Student’s t-test for samples of equal variance or unequal variance, depending on the result of the F-test. T-tests were corrected for multiple testing by Bonferroni method [19, 20]. Ratios of the metabolite levels were calculated by dividing average level of each metabolite in the developmental stages by the average level of metabolite of the previous developmental stage. In this way, the average of the 24 hpf group was divided by the levels of the early group and so on, resulting in the fold differences being an indication of what was happening from one developmental stage to the next. Hence, a value above one indicated that this metabolite increased from one developmental stage to the next, while a value below one showed that the metabolite decreased during the same time.

## Results

### Metabolic profiles of zebrafish during embryonic development

In total 33 samples (each containing 50 embryos) were collected and 81 metabolites were identified and relatively quantified in these samples. The samples were comprised of seven groups based on age (3/4 hpf, 4/5 hpf, 24 hpf, 48 hpf, 72 hpf and 96 hpf), whether they were artificially dechorionated with pronase and whether pigment was removed using PTU. The seven different groups have been summarised in Table 1.

**Table 1:** Sample groups used for modelling.

Group	Age of embryo (hpf)	Condition	Number of samples
1	3/4	Untreated	3
2	4/5	Untreated	5
3	24	Untreated	5
4	24	Treated with pronase to remove chorion	5
5	48	Treated with PTU to remove pigment	5
6	72	Untreated	5
7	96	Untreated	5

### Overview model of all samples

**Fig 2:**
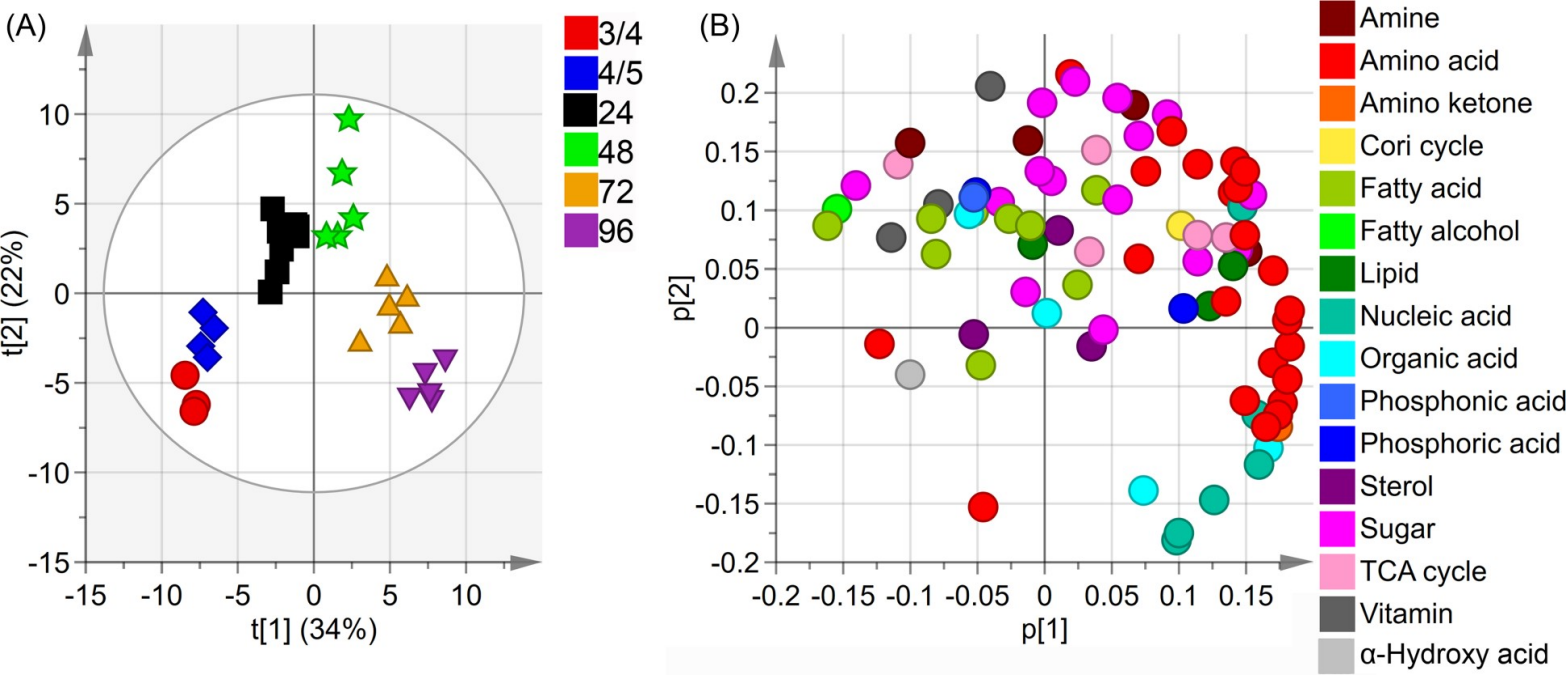
Score plot (t1/t2) for overview model after removal of sample 18_1. **2(A)**: The score plot showed the developmental stages. The first and second component explained 34% and 22% of the variation respectively. Different colours and shapes were used for the developmental samples: 3/4 hpf–red square, 4/5 hpf–blue rhombus, 24 hpf–black square, 48 hpf–green star, 72 hpf–orange pyramid and 96 hpf–purple triangle. **2(B)**: The loading plot showed the distribution of the identified metabolites. The metabolites were coloured according to metabolite class.

In the first PCA model generated there are a total of 6 groups. The 24 hpf group with chorion and the 24 hpf group without chorion were combined to form one group. Each group was given a class identity with the score plot coloured accordingly (Fig 2). The first two components explained 34% and 24% of the variation in the metabolites (the X matrix) and show clear clustering of the different classes, with the exception of one sample (preparation 18_1) which deviated from its class, see supporting information (S1 Fig).

The contribution plot for sample 18_1 showed that the levels of almost all metabolites were low, which may have been caused by an analytical problem. The contribution for sample 18_1 is available as supporting information (S2 Fig). This sample was classified as a true outlier and was therefore removed from any further modeling.

After sample 18_1 was removed, a new overview PCA model was calculated. This PCA model also explained approximately the same amount of variation (56%) in the first two components and the clustering of the different development stages were clear, see Fig 2.

The score plot showed that the metabolic profiles of the two early developmental groups (3/4 hpf and 4/5 hpf) were similar (CV-ANOVA for OPLS-DA model 3/4 hpf vs 4/5 hpf had p-value 0.01). Although the differences were significant, the variations were mainly associated with the 3/4 hpf group having lower levels of most metabolites. Since these two groups were sampled at approximately the same time and because the change between them was small enough to place the two groups together in the same quadrant, it was decided that when undertaking further analyses both groups would be treated as one, referred to as the early group. In the 24 hpf group the dechorionated samples and those still in their chorion overlap so could therefore also be regarded as one group in further analyses (CV-ANOVA for 24 hpf embryos in chorin vs dechorinated had p-value 0.36). The T-test showed that one of the 81 metabolites, spermidine (p-value 0.004, fold difference 1.5), differed significantly between those still in their chorion and dechorinated samples.

The loading plot for the overview PCA model showed that there were many metabolites contributing to the directional changes caused by the varying sample times. Lower levels of most metabolites were associated with the early group, followed by a rise in fatty acids and amines at 24 hpf and at 48 hpf the sugar levels were higher. Most amino acids were highest around 72 hpf to 96 hpf. At 96 hpf there was a rise in nucleic acids. Hence, metabolic fluctuation over the course of zebrafish development emerged.

### OPLS-DA models to identify metabolic differences

To study the metabolic fluctuations over time in the embryonic development of zebrafish, OPLS-DA models were calculated. The OPLS-DA models were used to compare the metabolic profile of one time point to the next. Hence, in the first OPLS-DA model the early stage embryos represented by the seven early samples (3/4 hpf and 4/5 hpf samples) were compared to the 24 hpf group consisting of ten samples. The second model compared the ten 24 hpf samples and the 48 hpf group consisting of five samples and so on. The quality of the resulting discriminant models have been summarised in Table 2.

**Table 2:** Quality of discriminant models.

Model	A	N	R^2^X	R^2^Y	Q^2^	P-value
Early vs 24 hpf	1+1+0	17	0.62	0.99	0.97	3.3*10^−9^
24 hpf vs 48 hpf	1+1+0	15	0.57	0.99	0.97	6.4*10^−8^
48 hpf vs 72 hpf	1+1+0	10	0.60	0.99	0.95	1.7*10^−3^
72 hpf vs 96 hpf	1+1+0	10	0.51	0.99	0.94	0.016

A is the number of components. N is the number of samples that the model is based on. R^2^X and R^2^Y are the cumulative variations explained in the metabolite and class-variable data respectively. Q^2^ is the cross-validated prediction estimate of class separation that shows how well samples are predicted by the model. The p-values were obtained using CV-ANOVA in SIMCA 14.0.

The OPLS-DA model between early stage embryos and 24 hpf embryos showed that 40 of the metabolites contributed to the model with a VIP measure above one. For the model comparing 24 hpf to 48 hpf 34 metabolites had a VIP measure above one. With regards to the model comparing 48 hpf and 72 hpf, 38 metabolites had a VIP measure above 1. The final model comparing 72 hpf to 96 hpf had 34 metabolites with a VIP measure above one. The findings in the OPLS-DA model were validated by t-test where we used Bonferroni correction, resulting in 47 out of the 81 metabolites being significantly different from at least one developmental stage to the next. The significant metabolites have been summarised into Table 3. Boxplots showing metabolite concentrations are available as supporting information (S3–S8 Figs).

**Table 3:** Significantly differing metabolites comparing one developmental stage to the next.

Metabolite	Class	Comparison							
		Early vs 24 hpf		24 hpf vs 48 hpf		48 hpf vs 72 hpf		72 hpf vs 96 hpf	
		p-value	Fold diff	p-value	Fold diff	p-value	Fold diff	p-value	Fold diff
Ethanolamine	Amine	3.6*10^−5^	1.2						
Putrescine	Amine	1.1*10^−9^	1.9	2.7*10^−4^	1.2				
Spermidine	Amine					5.0*10^−5^	0.4		
Urea	Amine	2.7*10^−7^	2.8						
Alanine	Amino acid	2.7*10^−7^	2.8						
Aspartate	Amino acid	9.9*10^−7^	0.2						
Glutamate	Amino acid			7.8*10^−10^	1.7	7.4*10^−5^	1.3		
Glutamine	Amino acid	1.6*10^−7^	2.7	4.9*10^−4^	1.3	3.9*10^−5^	2.1		
Histidine	Amino acid	1.7*10^−7^	2.6	3.1*10^−5^	1.4	1.8*10^−4^	1.7	1.1*10^−5^	2.0
Isoleucine	Amino acid	1.3*10^−7^	2.4						
Leucine	Amino acid	8.8*10^−7^	2.0						
Lysine	Amino acid	6.8*10^−7^	2.1						
Methionine	Amino acid	2.7*10^−6^	2.0	1.2*10^−6^	1.4				
Ornithine	Amino acid	6.6*10^−8^	2.4	2.5*10^−5^	1.4	8.9*10^−5^	1.6		
Phenylalanine	Amino acid	2.9*10^−12^	6.7	2.0*10^−11^	2.2			9.1*10^−5^	0.4
Proline	Amino acid	3.1*10^−4^	1.6						
Pyroglutamate	Amino acid	1.1*10^−5^	1.6	2.0*10^−9^	1.5	8.9*10^−6^	1.5		
Serine	Amino acid	1.5*10^−7^	2.2	3.0*10^−6^	0.6				
Threonine	Amino acid	4.3*10^−7^	2.1						
Tryptophan	Amino acid	5.9*10^−12^	5.3	1.1*10^−8^	1.9	1.4*10^−4^	1.4		
Tyrosine	Amino acid	8.4*10^−11^	4.3	8.6*10^−11^	3.1				
Valine	Amino acid	1.1*10^−7^	2.4						
Creatinine	Amino ketone	4.2*10^−5^	1.8	3.7*10^−9^	2.1	1.7*10^−4^	1.8	4.7*10^−4^	1.5
Lactate	Cori cycle	2.4*10^−6^	2.8						
Elaidate	Fatty acid	6.3*10^−6^	4.7						
n-Docosan-1-ol	Fatty alcohol					4.4*10^−4^	0.6		
n-Eicosan-1-ol	Fatty alcohol							2.3*10^−4^	0.6
Glycerol-3-phosphate	Lipid	4.0*10^−4^	1.4						
Adenosine-5-monophosphate	Nucleic acid			1.6*10^−4^	4.8				
Guanine	Nucleic acid	6.6*10^−7^	0.2			6.8*10^−5^	4.8	1.1*10^−4^	1.9
Hypoxanthine	Nucleic acid	2.1*10^−6^	0.2			4.2*10^−5^	3.8	1.0*10^−5^	2.1
Inosine	Nucleic acid	1.3*10^−9^	15.3						
Urate	Nucleic acid			2.0*10^−9^	42.7	3.7*10^−7^	5.4	1.8*10^−6^	1.9
4-amino-Butyrate	Organic acid			1.4*10^−12^	6.0	6.6*10^−6^	3.3		
Cellotriose	Sugar			9.0*10^−5^	6.5				
Fructose	Sugar	2.7*10^−4^	1.4	2.2*10^−4^	1.7				
Galactose	Sugar	6.2*10^−10^	3.9	1.3*10^−5^	1.9			8.4*10^−7^	0.5
Glucose	Sugar	4.0*10^−14^	28.0			7.0*10^−7^	0.3		
Glucose-6-phosphate	Sugar	1.1*10^−5^	2.6						
Maltose	Sugar			5.7*10^−4^	1.4				
Maltotrise	Sugar			4.7*10^−4^	3.5				
myo-Inositol	Sugar	1.0*10^−10^	2.8	4.6*10^−6^	1.5				
Ribose	Sugar	3.0*10^−9^	3.1						
Citrate	TCA cycle							7.0*10^−6^	0.4
alpha-Ketoglutarate	TCA cycle	5.0*10^−4^	1.8						
Malate	TCA cycle			2.8*10^−5^	1.6				
Ascorbate	Vitamin					4.2*10^−7^	0.4		

The p-values were calculated using two tailed Student’s t-tests and corrected for multiple testing by Bonferroni method. Fold differences indicated the average metabolite concentration, expressed per 50 embryos, at one developmental stage divided by the concentration at the previous stage. A value above one indicated that this metabolite increased from one developmental stage to the next, while a value below one showed that the metabolite decreased during the same time.

## Discussion

The results obtained in this study showed high similarity to those obtained in previous studies, where zebrafish metabolomic profiling at different developmental stages was also performed [7, 8, 10, 21]. Clear dynamic changes in metabolites were observed, which were found to be stage-specific and showed consistency such as a constant increase, constant decrease or no change during development, see boxplots available as supplementary information. The final PCA plot without the outlier showed clear clustering of stages with a directed shift from high levels of DNA building blocks at early stages to increased amino acid levels at intermediate stages and at later stages a shift towards elevated levels of metabolic waste products.

From the metabolomic results it was apparent that when comparing the early (3/4 and 4/5 hpf) embryos to 24 hpf embryos there was a change in metabolomic profiles. The early group had high levels of DNA and tRNA building blocks (guanine, hypoxanthine, but not inosine) present, whereas all identified amino acids and amino acid derivatives except aspartate increased by 24 hpf, indicating that as the embryos developed an increase in protein synthesis and levels of metabolites required for post-translational modifications occurred. This was similar to what was observed by Huang *et al*. who found the amino acid pool was supplied by the breakdown of the vitellogenin yolk protein by cathepsin at this developmental stage [7]. At 24 hpf, high levels of amino acids were accompanied by elevated levels of ribose (backbone of RNA), urea (waste product of amino acid deamination), glucose and glucose-6-phosphate (metabolic fuel) as well as galactose, fructose and myo-inositol (readily converted to glucose), which indicated an increase in energy demand. Alpha-ketoglutarate was found to increase from the early developmental stage to 24 hpf. This is an intermediate in the TCA cycle that plays an important role in the rate determination of TCA cycle turnover [22], presenting another sign of an increase in energy demand. Additionally, increased levels of creatinine (waste product of creatine phosphate) and lactic acid were also detected, which indicated that the muscles were starting to contract. Putrescine was also found to increase from early development to 24 hpf. Polyamines such as putrescine are produced by dead cells but putrescine is also required in small amounts for cell growth, cell signalling and cell division [23]. Lipid associated metabolites such as ethanolamine (second most abundant head of lipids), elaidic acid (fatty acid that can be incorporated in lipid membranes [24]) and glycerol-3-phosphate (starting material for glycerolipid synthesis) also increased between the early developmental stage and 24 hpf.

When comparing the metabolic profiles between 24 and 48 hpf embryos, the increased levels of creatinine remained. Amino acids and amino acid derivatives increased (e.g. glutamate, glutamine, histidine, methionine, ornithine, pyroglutamate, phenylalanine, tryptophan and tyrosine) but serine decreased, indicating that the protein synthesis increased between 24 and 48 hpf. Most of the identified sugars increased between these two developmental stages, i.e. galactose, fructose, cellotriose, maltose, maltriose, and myo-inositol, while the high levels of glucose remained, which indicated a continuous increase in energy demand and continuous growth promotion [25]. The increased levels of adenosine-5-phosphate indicated that energy charge is low and that increasing amounts of ATP has been used in biochemical processes. Malate had increased between 24 and 48 hpf, further indicating high energy demand. Moreover, butyric acid which inhibits histone deacetylases (HDACs) that silence promoters was elevated indicating that there was an increase in gene transcription [26]. Putrescine continued to increase between 24 to 48 hpf as a sign of increased cell death, cell growth, cell signalling and/or cell division [23]. Increased levels of uric acid showed increased nitrogen metabolism. LeMoine *et al*. have also found embryos between 30 and 48 hpf to have elevated ammonia excretion [27], which was further supported by the reduced urea levels in 48 hpf zebrafish embryos. Together these findings suggest that the liver was starting to function to remove waste products between 24 and 48 hpf.

When comparing the metabolic profiles of 48 to 72 hpf creatinine still increased significantly. The majority of amino acids continued to rise in concentration from 48 to 72 hpf (e.g. glutamate, glutamine, histidine, ornithine and tryptophan), in addition to pyroglutamic acid indicating that many post-translational modifications were still occurring. Glucose levels were reduced, which may have been caused by the action of insulin (as the endocrine pancreas is formed at 72 hpf) or as a consequence of high turnover. The elevated levels of glutamine suggested that at 72 hpf glutamine catabolism may have been occurring to provide additional energy. Additionally, ornithine levels were high suggesting that the urea cycle was still highly active at 72 hpf. Moreover, butyric acid concentrations were continuing to rise, indicating an increase in gene transcription [26]. This was further strengthened by the fact that the levels of DNA and tRNA building blocks guanine and hypoxanthine increased between 48 and 72 hpf. Uric acid was significantly elevated at 72 hpf. Interestingly, in zebrafish uric acid is converted to allantoin by the uricase enzyme. In humans uric acid is the final product of purine metabolism, as the uricase enzyme has been lost during evolution in humans [28]. Allantoin (product of uric acid oxidation) was also found in higher levels, however not significantly higher than at 48 hpf.

Several metabolites decreased significantly between 48 and 72 hpf including spermidine and its biochemical precursor putrescine. Their common precursor is the aforementioned elevated ornithine [29]. The polyamines putrescine and spermidine have been reported to be important for viability, proliferation and differentiation of cells [30]. Ascorbate and *n*-Eicosan-1-ol were also found to decrease from 48 to 72 hpf. Ascorbate is important for the maintenance of cellular energy metabolism in zebrafish [31].

When comparing the metabolic profiles at 96 hpf to 72 hpf, the levels of creatinine, guanine, hypoxanthine, uric acid and histidine remained high. The increased levels of guanine, hypoxanthine, serine (important for biosynthesis of purines/pyrimidines) indicated that there was again an increase in DNA synthesis. However, as citric acid levels were reduced, but uric acid (breakdown product of purines) levels remained high, it is possible that purines were also being catabolised to meet the high energy demands during development.

Several metabolites were also found to decrease significantly between 72 and 96 hpf including galactose, phenylalanine and *n*-Docosan-1-ol. Several amino acids plateaued between 72 and 96 hpf whereas phenylalanine decreased significantly. Tufi *et al*. also found neurotransmitter precursors tryptophan, tyrosine and phenylalanine to peak around three days post fertilisation (72 hpf) and attributed this to the change in metabolism that hatching involves [32]. By 96 hpf most sugars had decreased, out of which galactose decreased significantly, indicating that the energy demand was reduced.

Throughout the different developmental stages it was apparent that creatinine levels remained high and increased. This was also observed in metabolomic studies carried out by Soanes *et al*., 2011 [10]. The reason for high creatinine levels is thought to be a consequence of the high energy demand required during the development of the embryo into a swimming larva. Additionally, branched amino acids were found to be present at constant levels throughout the developmental process, similar to the studies carried out by Soanes *et al*., 2011 [10]. There was a gradual increase in many of the identified amino acids, supporting the findings of Huang *et al*. [7].

## Conclusions

The metabolic stability and the viability of the embryos suggest that 72 hpf is the most suitable time point for use of zebrafish as a model system in applications including for example vertebra development, toxicology and genotype studies. One metabolite differed significantly in concentration between dechorinated embryos and those still in their chorion. This was spermidine which was reported to be important for viability, proliferation and differentiation of cells. The time period between the earliest developmental stage and 24 hpf was characterized by significant metabolic changes where there was a marked increase in building blocks, i.e. amino acids and nucleic acids as well as a significant increase in energy metabolites i.e. sugars and TCA cycle intermediates. The same pattern was observable between 24 hpf and 48 hpf, as well as 48 hpf and 72 hpf, however with fewer and fewer significant differences. The smallest significant differences were observed between 72 and 96 hpf. The time point between 72 and 96 hpf was characterized by an increased DNA synthesis, reduced energy demand and a peak in neurotransmitter precursors around 72 hpf. Interestingly, the levels of free fatty acids and sterols remained stable between the investigated developmental stages while fatty alcohols decreased over the studied time period. We propose further studies to investigate the role of fatty acids and alcohols in zebrafish development.

## Supporting information

S1 Data Contains the Zebrafish metabolomics data. (XLSX)

S1 Fig Score plot (t1/t2) for the overview PCA model. (DOCX)

S2 Fig Contribution plot for sample 18_1 compared to the center of the model. (DOCX)

S3 Fig Box plot–Significant differences between several developmental stages. (DOCX)

S4 Fig Box plot–Significant difference from Early to 24 hpf. (DOCX)

S5 Fig Box plot–Significant difference from 24 hpf to 48 hpf. (DOCX)

S6 Fig Box plot–Significant difference from 48 hpf to 72 hpf. (DOCX)

S7 Fig Box plot–Significant difference from 72 hpf to 96 hpf. (DOCX)

S8 Fig Box plot—No significant differences. (DOCX)
