# Supplementary material for: Metabolic profiling of zebrafish embryo development from blastula period to early larval stages
Source: PLoS One. 2019 May 14;14(5):e0213661. doi: 10.1371/journal.pone.0213661 (PMC6516655; doi:10.1371/journal.pone.0213661)
Supplement: S1 Fig — (DOCX) [file pone.0213661.s002.docx]

# Supporting information


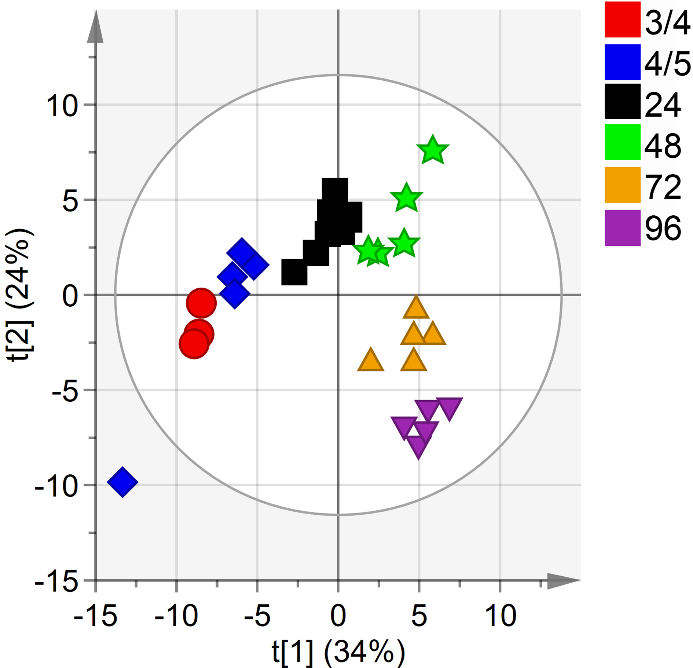


**S1 Fig. Score plot (t1/t2) for the overview PCA model.** The first and second component explained 34% and 24% of the variation respectively. Different colours and shapes were used for the developmental samples: 3/4 hpf – red square, 4/5 hpf – blue rhombus, 24 hpf – black square, 48 hpf – green star, 72 hpf – orange pyramid and 96 hpf – purple triangle.
