# Supplementary material for: Metabolic profiling of zebrafish embryo development from blastula period to early larval stages
Source: PLoS One. 2019 May 14;14(5):e0213661. doi: 10.1371/journal.pone.0213661 (PMC6516655; doi:10.1371/journal.pone.0213661)
Supplement: S2 Fig — (DOCX) [file pone.0213661.s003.docx]

# Supporting information


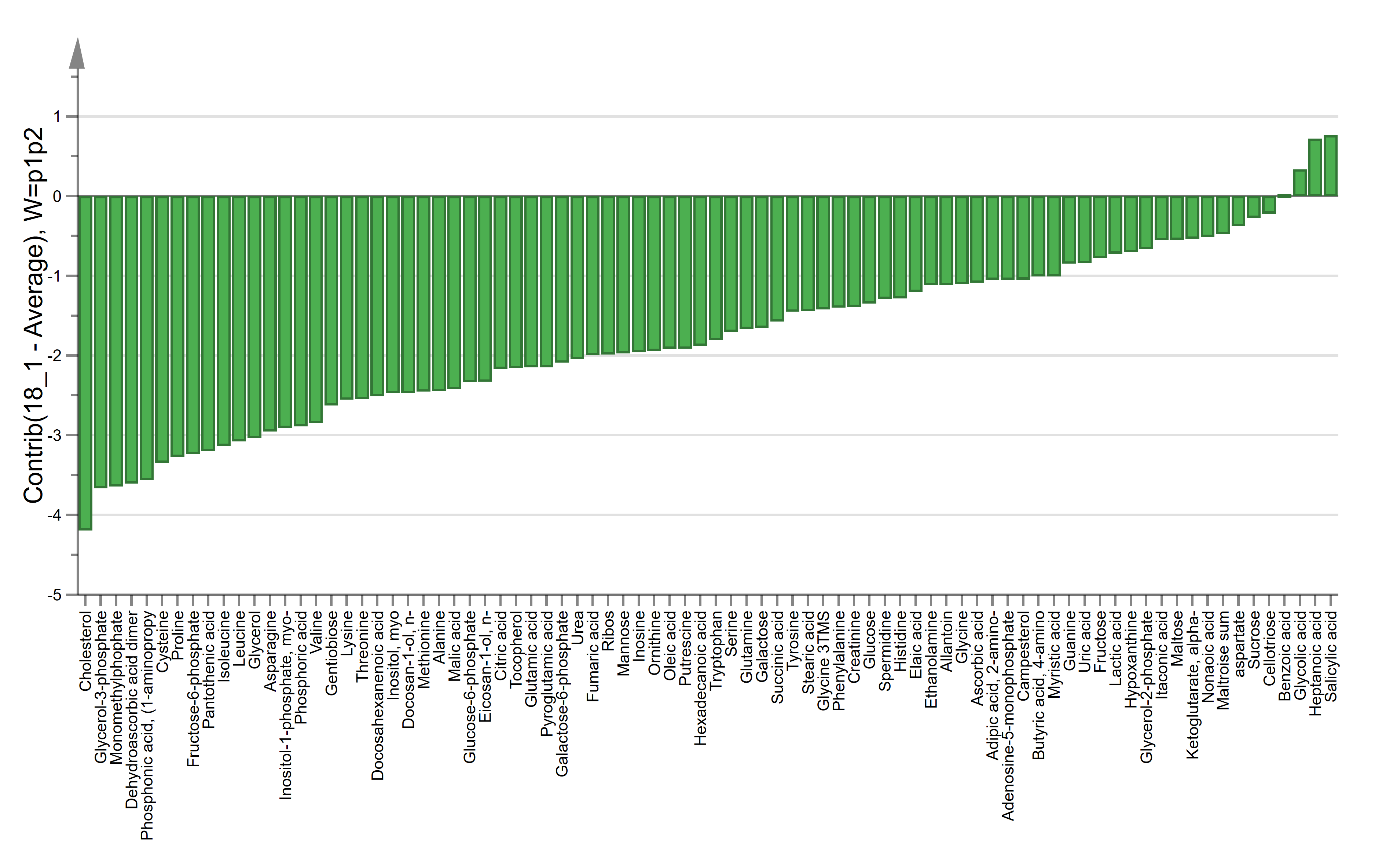


**S2 Fig. Contribution plot for sample 18_1 compared to the center of the model.** The majority of the metabolites were found in lower levels in this sample, indicating that there may have been analytical problems with this particular sample.
