# Supplementary material for: Metabolic profiling of zebrafish embryo development from blastula period to early larval stages
Source: PLoS One. 2019 May 14;14(5):e0213661. doi: 10.1371/journal.pone.0213661 (PMC6516655; doi:10.1371/journal.pone.0213661)
Supplement: S6 Fig — (DOCX) [file pone.0213661.s007.docx]

# Supporting information


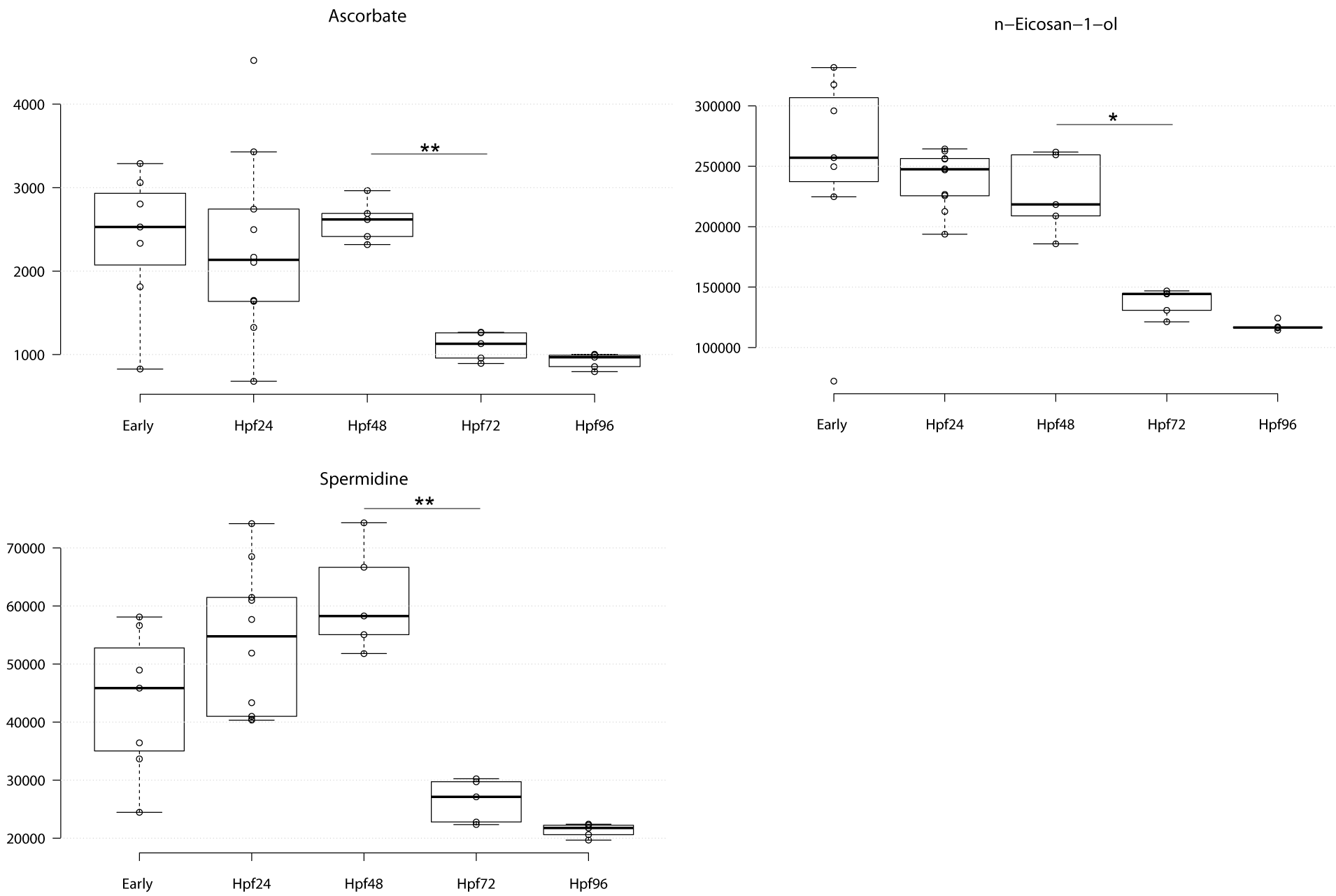


**S6 Fig. Box plot – Significant difference from 48 hpf to 72 hpf**. These three metabolites displayed significant differences between 48 hpf to 72 hpf. Significance of the differences were indicated by asterisks where three asterisks indicated a p-value smaller than 1.0*10^–6^, one asterisk a p-value greater than 1.0*10^–4^ and two asterisks a p-value in between. The center lines show the medians. The box limits indicated the 25th and 75th percentiles as determined by R software. The whiskers extended 1.5 times the interquartile range from the 25th and 75th percentiles. All data points were plotted as open circles.
