# Supplementary material for: Metabolic profiling of zebrafish embryo development from blastula period to early larval stages
Source: PLoS One. 2019 May 14;14(5):e0213661. doi: 10.1371/journal.pone.0213661 (PMC6516655; doi:10.1371/journal.pone.0213661)
Supplement: S8 Fig — (DOCX) [file pone.0213661.s009.docx]

# Supporting information


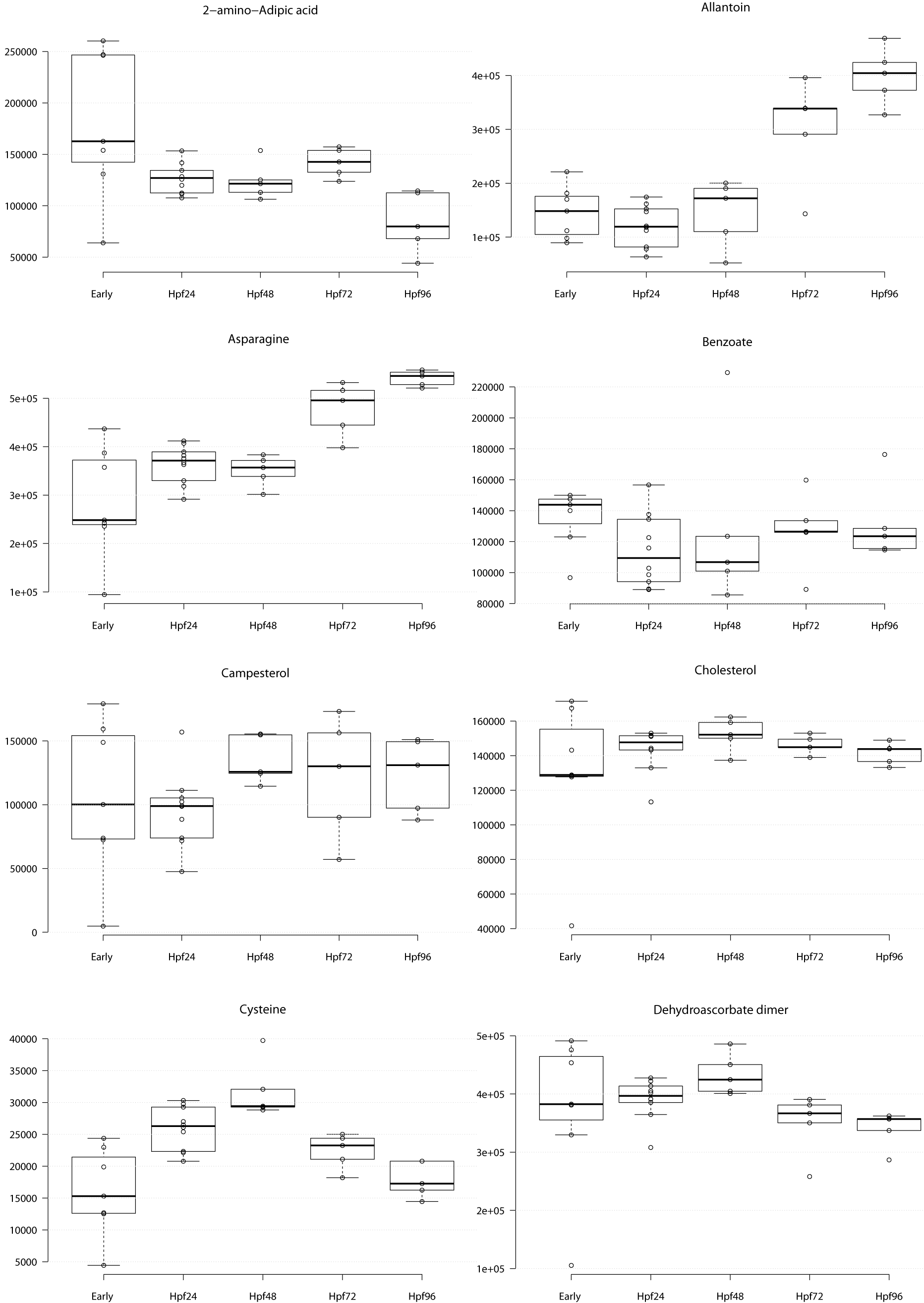


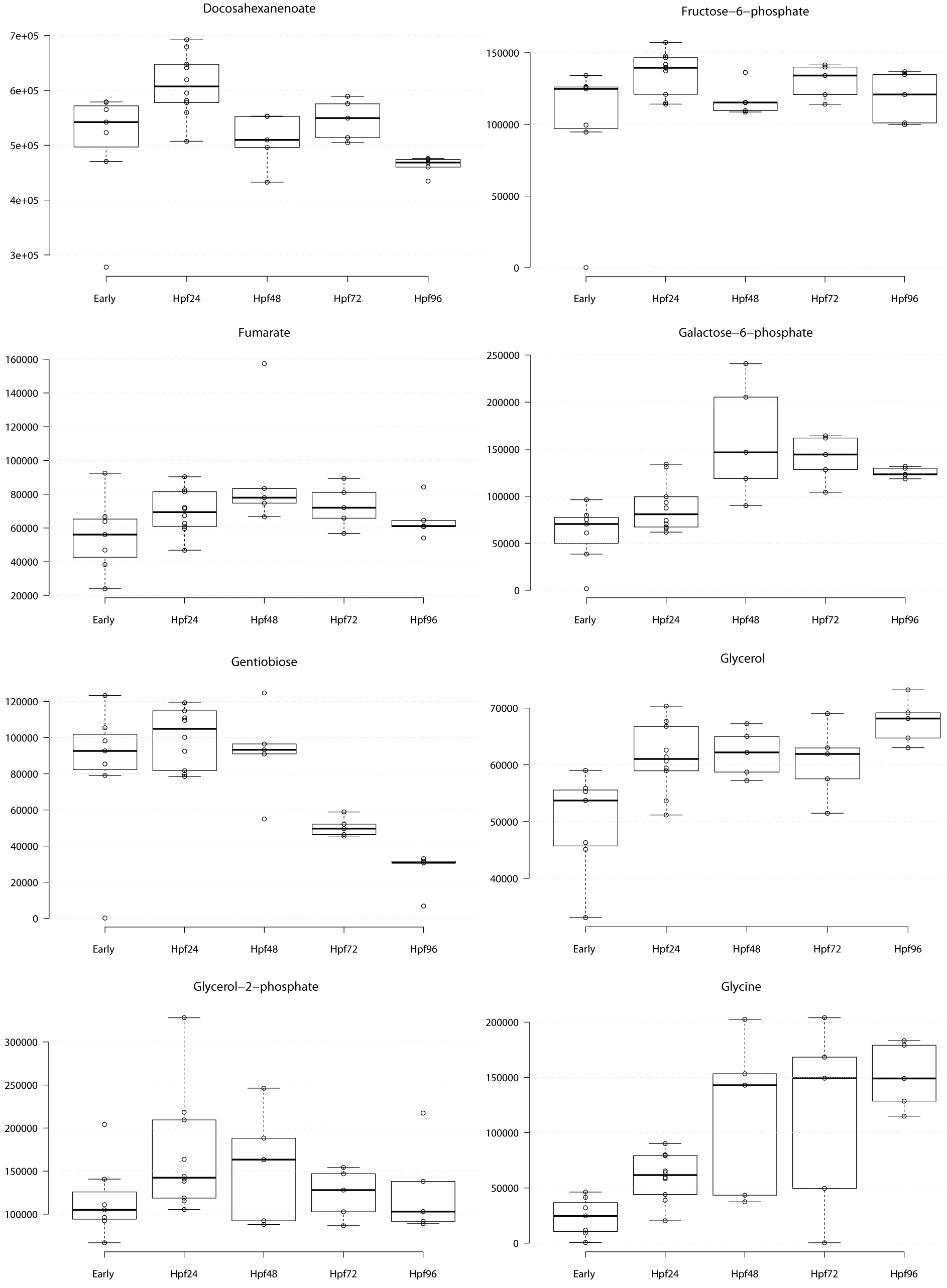


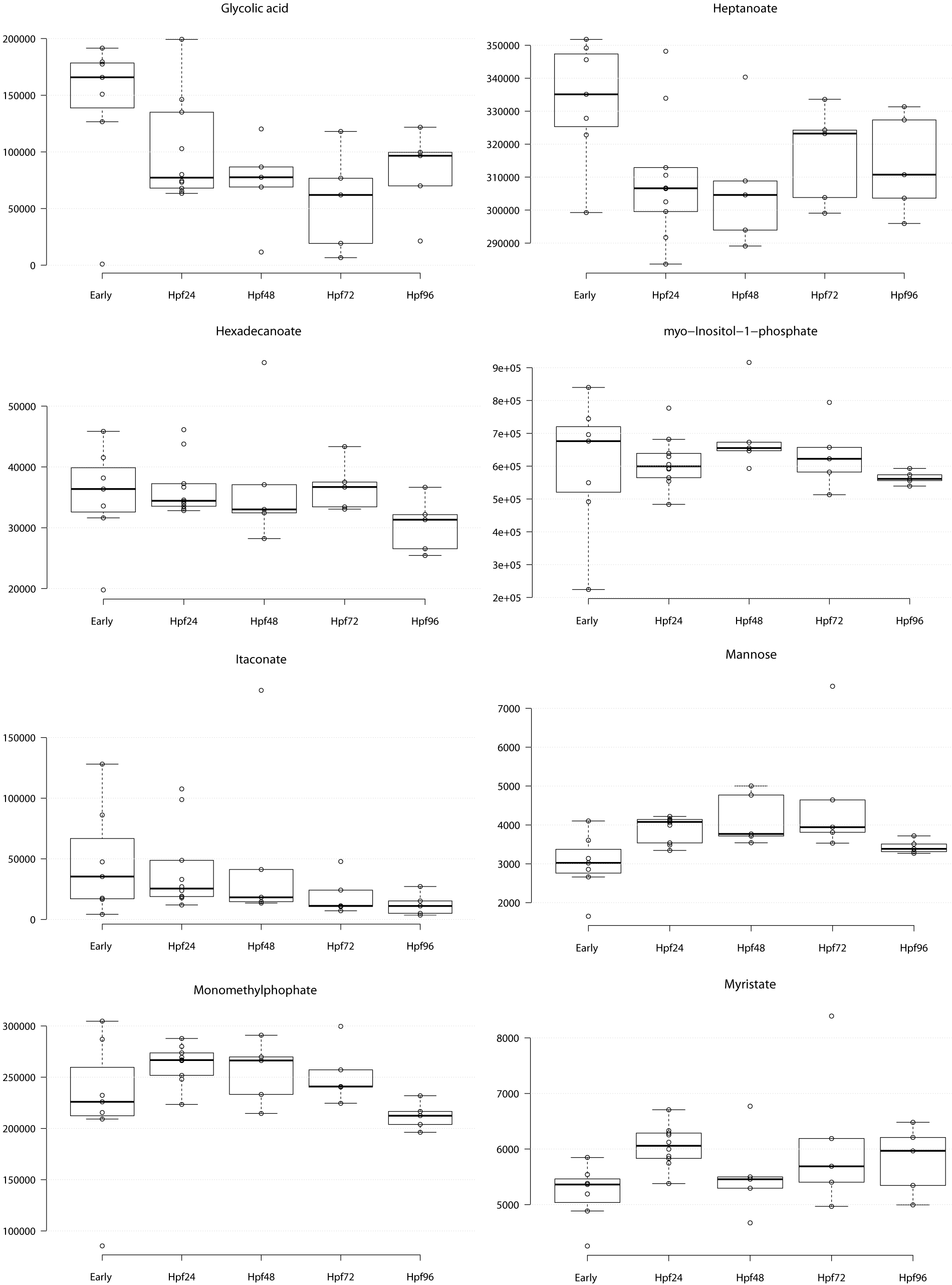


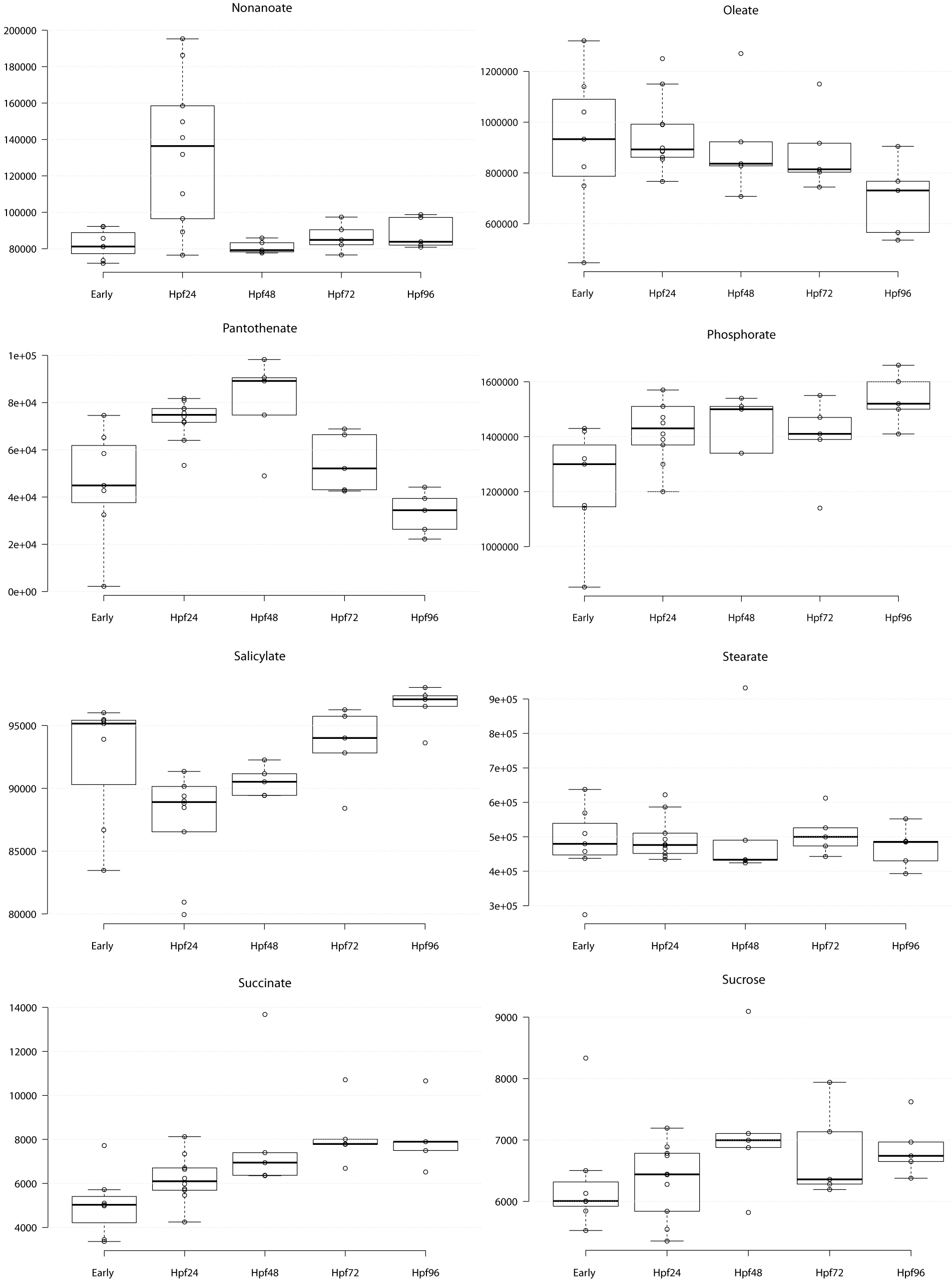


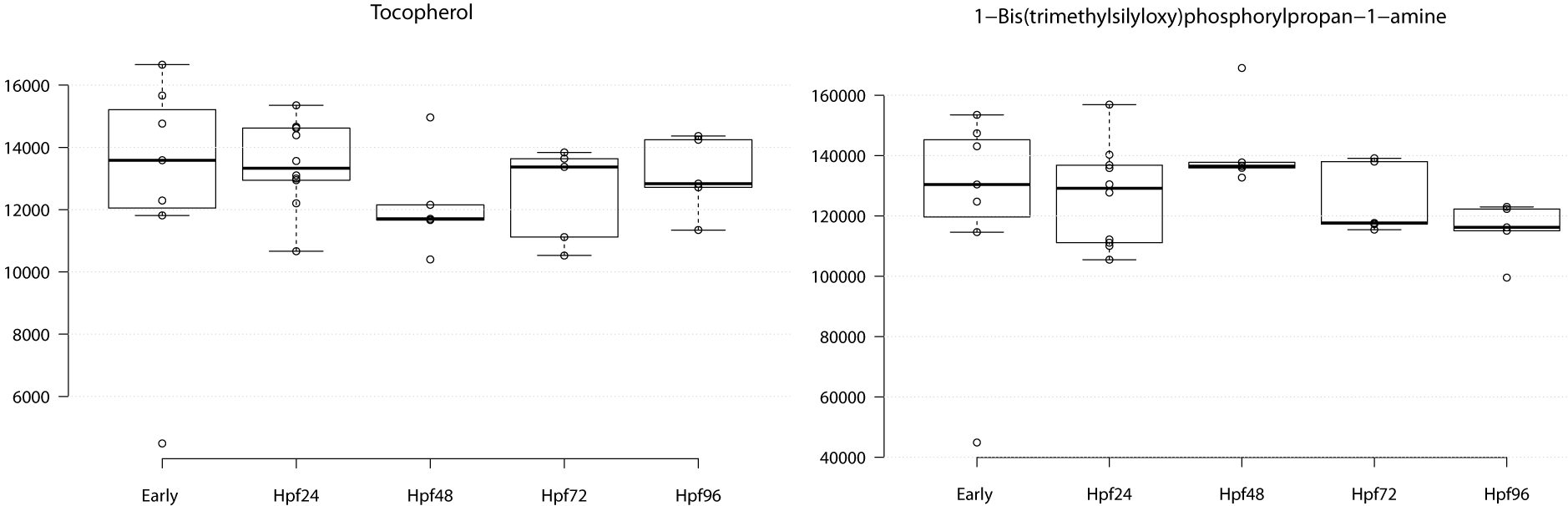


**S8 Fig. Box plot - No significant differences**. These 37 metabolites did not display any significant changes in concentration form one developmental stage to another. The center lines show the medians. The box limits indicated the 25th and 75th percentiles as determined by R software. The whiskers extended 1.5 times the interquartile range from the 25th and 75th percentiles. All data points were plotted as open circles.
